# A Report of a Symptomatic Progressive Myeloma during Pregnancy and Postpartum Period from Asymptomatic State

**DOI:** 10.3390/hematolrep15020031

**Published:** 2023-05-05

**Authors:** Gehad Elgabry, Lydia Spencer, Hisam Siddiqi, Soumya Ojha, Farooq Wandroo

**Affiliations:** 1 Department of Haematology, Sandwell and West Birmingham Hospitals NHS Trust, West Bromwich B71 4HJ, UK; 2 Department of Haematology, University Hospital Birmingham NHS Trust, Birmingham B15 2GW, UK

**Keywords:** multiple myeloma, myeloma, pregnancy

## Abstract

Multiple myeloma is a plasma cell malignancy that is most commonly observed in males in the sixth and seventh decade of life. The clinical scenario of multiple myeloma with concurrent pregnancy is considered to be very rare. We detail here the case of a young female with known IgG kappa multiple myeloma who was found to have a steady elevation of her IgG kappa paraprotein during pregnancy and symptomatic progression in the postpartum period. She delivered a healthy baby at 40 weeks gestation. We present a review of all reported cases of known multiple myeloma progressing during pregnancy and in the postpartum period, the treatments given, and their outcomes. The report also provides suggestions for diagnosis and management of myeloma during pregnancy in order to have an outcome of successful uncomplicated pregnancy with healthy offspring.

## 1. Introduction

Multiple myeloma accounts for 15% of all blood cancers and 2% of all cancers generally in UK [1]. Myeloma in pregnancy is a rare occurrence as the median age of myeloma diagnosis is around the sixth and seventh decade of life and is more commonly observed in males [2,3]. The first case of myeloma in pregnancy was reported in 1965 [4], and since then there have been fewer than fifty cases reported worldwide [5]. Although only three percent of cases are diagnosed below the age of forty [1], myeloma is now increasingly being diagnosed in younger ages and during pregnancy due to better screening and awareness [6]. We report here a case of successful outcome of pregnancy in a young patient of IgG kappa myeloma and a review of literature of multiple myeloma progressing during pregnancy and in the postpartum period.

## 2. Case Presentation

This is a case of a 35-year-old female with a known diagnosis of IgG kappa multiple myeloma. She was diagnosed with multiple myeloma in 2016 when she presented following a fall with back pain, with magnetic resonance imaging (MRI) showing multiple pathological vertebral fractures. Laboratory findings showed monoclonal IgG kappa paraprotein level of 42 g/L, hemoglobin 12.7 g/dL, creatinine 66 mmol/L, calcium 2.3 mmol/L, Beta-2 microglobulin of 3.8 mg/L, lactate dehydrogenase (LDH) levels of 104 g/L, and serum albumin levels of 28 g/L. Serum free light assay was not available at presentation. Bone marrow biopsy showed 80% plasma cell infiltration. Her revised international staging system (R-ISS) stage showed R-ISS stage-II. She subsequently underwent three vertebroplasties and was treated with eight cycles of velcade, cyclophosphamide/thalidomide, and dexamethasone (VCD/VTD) chemotherapy, achieving a very good partial response, following which she had an autologous stem cell transplant (ASCT) in 2017. She was regularly monitored with three monthly light chain assay and biochemical assessment. She had commenced once monthly zoledronic acid infusions at the time of induction treatment, which was reduced to every three months after autologous stem cell transplantation to complete 3 years.

**Figure 1:**
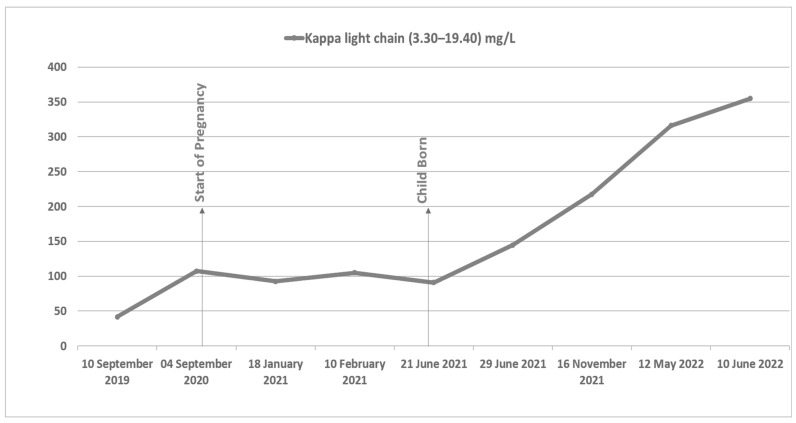
A graph of Serum Kappa light chain levels from 10 September 2019 (12 months prior to onset of pregnancy) to 10 June 2022 (12 months post-delivery).

Four years post ASCT in 2020, a slow kappa light chain increase was noted (Figure 1). During this time—and unbeknown to the patient—she had become pregnant. Ultrasound imaging confirmed a single live fetus with no fetal anomalies at 22 + 6 gestation.

A multi-disciplinary team meeting (MDT) was held, and it was agreed that she did not require any active treatment of her myeloma at that time as she had normal bone marrow function, normal calcium, and renal function and no new lytic lesions on imaging.

Her serum free light chain assay had a kappa/lambda ratio of 7.7 mgs/L (kappa 107.2 mgs/L, Lambda 13.8 mgs/L). Her bone marrow had only 30% plasma cells; hence, she did not fit SLIM CRAB criteria for treatment. Therefore, a plan was made for regular clinical observation. Furthermore, she was given low molecular weight heparin in line with Royal College of Obstetricians and Gynecologists guidelines based on her risk profile [7].

Throughout the course of the pregnancy, her IgG kappa paraprotein only increased slightly from 28 g/L at the onset of pregnancy to 34.2 g/L at childbirth in the face of a steady elevation of kappa light chains at 100 mgs/L. Her serum chemistry, hematinics, and full blood count remained normal. She delivered a healthy baby girl via unassisted vaginal delivery at 40 weeks gestation.

**Figure 2:**
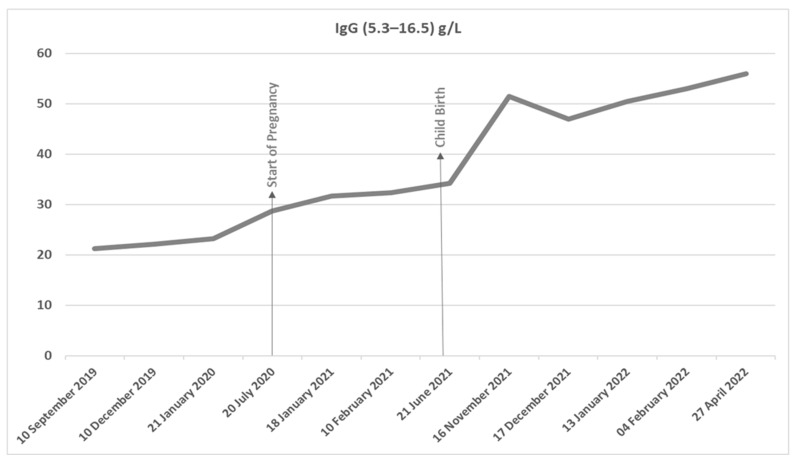
A graph of Serum Monoclonal IgG kappa paraprotein levels from 10 September 2019 (12 months prior to onset of pregnancy) to 27 April 2022 (10 months post-delivery).

Following delivery, both IgG kappa monoclonal protein levels and serum kappa light chain levels started to rise more markedly (Figure 2). At eleven months post-partum, she was noted to be anemic (Hb 99 g/L), with high serum IgG kappa levels (53.0 g/L) and elevated serum Kappa light chain (316.2 mg/L) (Figure 1). Repeat bone marrow biopsy showed almost complete replacement with dense infiltrate of CD138 positive plasma cells in keeping with marked progression of myeloma. Repeat MRI spine at relapse when compared with presentation MRI at diagnosis (which had shown multiple vertebral collapses from T11 to L1 spinal vertebrae) showed no new lytic lesions or new vertebral collapse, and both her renal function and calcium remained normal. She was commenced on third-line anti-myeloma treatment with lenalidomide, dexamethasone, and ixazomib, to which she had a suboptimal response. She has now commenced fourth-line treatment with isatuximab, pomalidomide, and dexamethasone—following which she will be referred to the stem cell transplant service for consideration of second ASCT.

## 3. Discussion

This is one of the few cases in the literature that report a relapse or progression of known multiple myeloma in patients who became pregnant. Of the 46 cases (including this case) reported, only three other cases reported relapse of myeloma in pregnancy or the postpartum period (Table 1).

Among the previously reported cases is a case of a patient with monoclonal gammopathy of undetermined significance (MGUS) diagnosed 6 years prior to pregnancy. During the pregnancy, she remained asymptomatic relative to CRAB symptoms (smoldering myeloma) and then developed symptomatic multiple myeloma in the post-partum period [8].

Khot et al. presented a similar case of a patient who was in complete remission for 5 years following ASCT but whose myeloma progressed during pregnancy. As the patient had normal biochemistry except for asymptomatic anemia and no bone lesions, she did not receive any treatment during pregnancy and was commenced on Lenalidomide and Dexamethasone post-cesarean section [9].

Finally, Brisou et al. reported what may be the earliest case report of a patient whom after 10 years in remission post ASCT developed new back pain and anemia associated with rising kappa light chain levels at 7 months pregnant [10]. MRI showed minimal lesions of both the skull and pelvis. She received no anti-myeloma treatment in pregnancy and delivered a healthy baby at 37 weeks gestation via cesarean. Four months post-delivery, she received third-line chemotherapy with five cycles of bortezomib, cyclophosphamide, and dexamethasone, followed by salvage ASCT.

All four cases reported births of healthy infants. Magen et al. found that between 1965 and 2020 of the 42 cases reported who continued with their pregnancy, 40 of these had healthy babies irrespective of the administration of treatment versus observation during pregnancy [5]. Interestingly, the two reported cases that ended in fetal demise were reported to have had no anti-myeloma treatment before or during the pregnancy [5]. Among eight patients who had been diagnosed before conception, five of them had active myeloma, suggesting that disease does not hamper conception and the need for continued counseling in relation to pregnancy prevention, especially if the women are on immunomodulatory drugs such as lenalidomide. In the case reported by Magen et al. presenting with hypertension and acute renal failure, the patient was initially thought to have pregnancy-related complications and infection and was diagnosed as having tubulointerstitial disease on renal biopsy. It was only at 25 weeks (10 weeks after presentation) that high kappa light chains were detected, suggesting a need for educating our physicians and obstetricians to think about myeloma in such scenarios. Further in their extensive report, of the thirty-three patients diagnosed during pregnancy, the majority, i.e., twenty-six, were diagnosed during the second, and only seven in the first, trimester [5]. This may suggest a causal relationship between the evolution of myeloma and pregnancy progression and a role of modifications in hormonal and cytokine milieu during pregnancy. It could be argued that, in the case we are talking about, it has been four years since her autologous transplant therefore her myeloma may have naturally progressed—irrespective of pregnancy—as the median time to progression after autologous transplant is around 42 months [11]. However, it is equally important not to discard the effects of pregnancy on myeloma progression due to changes in hormonal and cytokine milieu. Bommert et al. observed that levels of Interleukin-6 (IL-6) as well as Insulin Like Growth Factor (IGF-1) were increased during pregnancy, indicating an immunological shift of T- Helper 1/T helper- 2 cells tilting the balance towards increase in T- Helper 2 cells [12]. The role IL6 as a growth factor in multiple myeloma is well recognized. It could be therefore hypothesized that the pregnant state creates a permissive bone marrow environment for the progression of myeloma. IL6 is a paracrine factor and is produced by cells of the myeloid lineage. The up regulation of IL6 by myeloid cells produced another key growth factor for multiple myeloma, a proliferation inducing ligand (APRIL) [13]. The first clinical trials did not demonstrate a clear benefit, but despite these setbacks hopes regarding IL-6 antagonism are still high and trials are ongoing [14]. Similarly, Tavani et al., in 1997, assessed reproductive factors and the risks of developing lymphoma and myeloma in a case-controlled study. The study found that the immunological changes that occur in early pregnancy are likely mechanisms for the protection of patients from the risk of developing Hodgkin’s lymphoma but did not protect against myeloma or non-Hodgkin’s lymphoma [15]. Danel et al. have shown myeloma cells to have either estrogen or progesterone receptors and that previous in vitro studies have shown that the presence of an estrogen receptor modulator reduced myeloma cell proliferation [16]. Ozerova and colleagues demonstrated through mice models that estrogen may enhance the immunosuppressive effects of the myeloid-derived suppressor cells (MDSCs), which are found in the myeloma tumor microenvironment and therefore may play a role in the progression of myeloma in pregnancy [17,18]. Tavani et al., in 1997, assessed reproductive factors and the risks of developing lymphoma and myeloma in a case-controlled study. The study found that the immunological changes that occur in early pregnancy are likely mechanisms for the protection of patients from the risk of developing Hodgkin’s lymphoma but did not protect against myeloma or non-Hodgkin’s lymphoma [15].

Further, it is interesting to observe in the large series of cases reported by Magen et al. that the majority (ten patients) had IgG lambda myeloma, while an equal number had light chain myeloma and one case had plasma cell leukemia [5]. The increased incidence of light chain myeloma has been seen previously in a large case series of younger myeloma patients [19]. The majority of these had Durie–Salmon three or ISS stage 1 disease. This is consistent with similar reports of low-risk disease in non-pregnant myeloma patients diagnosed below the age of fifty years [19].

Physicians can face a dilemma as to what treatments are safer during pregnancy. In cases reported by Magen et al., the patients who required treatment majority were given either dexamethasone or cyclophosphamide [5]. Both corticosteroids and cyclophosphamide are relatively safe during pregnancy, although high doses of corticosteroids can cause fetal malformations, especially if given during the first trimester. They can also cause maternal and obstetric complications in the later part of the pregnancy. Since prednisolone is metabolized by the placenta, it is thought to be a safer corticosteroid during pregnancy [20,21,22,23]. Cyclophosphamide is the main chemotherapy agent used in nearly all cases where chemotherapy was indicated with no deleterious effects on the fetus; however, most had it during the second and third trimester [4,24,25]. Of the immunomodulatory drugs, proteasome inhibitors have all been shown to cause embryo-fetal toxicity in animals and hence are not recommend during pregnancy as there are no data from animal studies on the use of anti-CD38 monoclonal antibodies during pregnancy [5].

It is encouraging to see that fetal complications were rare with only five out of forty-five pregnancies ending in fetal loss [5]. However, multiple myeloma may contribute to worse pregnancy outcomes in a number of different ways. Firstly, anemia is a common complication of multiple myeloma; separately, anemia frequently occurs in normal pregnancy due to increased blood volume and iron deficiency. Therefore, we can expect patients in whom pregnancy and multiple myeloma coincide to potentially be predisposed to severe multifactorial anemia. This has been associated with premature birth and low birth weight [26]. Another clinical concern is hypercalcemia occurring as a metabolic complication of multiple myeloma. This can produce vomiting and dehydration, which, when combined with hyperemesis in pregnancy, may significantly worsen the severity of symptoms and necessitate urgent treatment [27]. Hypercalcemia has also been associated with increased rates of pre-eclampsia in some reports [28]. Furthermore, bone pain and vertebral fractures appear to be a common feature of multiple myeloma in pregnancy [29]. It is also important to educate obstetricians and physicians looking after pregnant patients to consider a myeloma screen when complications such as renal impairment, anemia with normal hematinics, and unusual bone pain or symptoms of hypercalcemia such as persistent vomiting are present.

## 4. Conclusions

This is another case to add to the existing literature on the rare occurrence of myeloma in pregnancy, and in which pregnancy may have played a causal role in disease relapse. This case highlights the possible management strategy of waiting and watching pregnant patients with myeloma but also highlights the need for vigilant monitoring in both pregnancy and the post-partum period.

There is currently insufficient evidence to suggest that pregnancy is contraindicated in patients with multiple myeloma. The cases reported suggest that patients with child-bearing potential can have successful pregnancies despite multiple myeloma. However, as evidenced in this and other case reports, the propensity for myeloma to progress during pregnancy adds to the ethical, emotional, and therapeutic challenges patients and healthcare professionals face. Furthermore, due to the rarity of this presentation and the unfeasibility of conducting ethical trials of anti-myeloma treatments in pregnant women, the data regarding therapeutic options remain anecdotal.

We suggest that these patients should be monitored closely, with clinical and laboratory checks on both mother and fetus, and a close liaison between hematologist and obstetrician is mandatory. The mother’s hematinics must be replaced as appropriate, and the mother must be encouraged to keep well hydrated. The mother must be given thromboprophylaxis in accordance with Royal College of Obstetric guidelines. Attention should be paid to early symptoms of preeclampsia, hypercalcemia, and bony symptoms and appropriately evaluated. The baby and mother should be closely monitored in the first few months postpartum, especially if the mother has had anti-myeloma treatment during pregnancy.

Although large majority of patients can be monitored without treatment, some patients may need treatment during pregnancy. The aim should be to use minimal treatment to control the disease and symptoms and to take the pregnancy to the age of fetal viability. Once the baby is delivered, full-dose treatment can be instituted. It is also important to keep in mind that myeloma, although not active, may play a role in inducing complications during pregnancy.

## Figures and Tables

**Table 1:** Reported cases of disease relapse or progression during pregnancy or postpartum period.

Reference	Relapse/Progression	Characteristic of Relapse	Treatment Given	Foetal Outcome	Maternal Outcome
J.C. Lee et al. [8]	Symtomatic relapse in postpartum period	Plasmacytosis in bone marrow, new bone lytic lesions, renal failure, hypercalcemia, and Paraprotein 41.1 g/L,	High-dose oral dexamethasone, four plasma exchanges, allopurinol, pamidronate, and renal dialysis. Vincristine, adriamycin, and dexamethasone chemotherapy planned.	Healthy	Deceased following intracerebral hemorrhage, 1 month after progression in postpartum period.
Khot et al. [9]	Symtomatic progression during pregnancy	Rising serum free light chains, new bone marrow plasmacytosis of 20%, and anemia.	Lenalidomide and dexamethasone post cesarean-section. Plan to proceed to further high dose therapy and allogenic transplant at progression.	Healthy	Alive at 5 years since relapse when reported
G. Brisou et al. [10]	Symtomaticrelapse during pregnancy	Back pain, rising kappa light chains, symptomatic anemia, new lytic lesions inskull, and pelvis on MRI.	Bortezomib, cyclophosphamide, and dexamethasone post-delivery.	Healthy	Alive at 19 months since relapse when last reported following second ASCT.

## Data Availability

Not applicable.
